# Improving platelet‐RNA‐based diagnostics: a comparative analysis of machine learning models for cancer detection and multiclass classification

**DOI:** 10.1002/1878-0261.13689

**Published:** 2024-06-17

**Authors:** Maksym A. Jopek, Krzysztof Pastuszak, Michał Sieczczyński, Sebastian Cygert, Anna J. Żaczek, Matthew T. Rondina, Anna Supernat

**Affiliations:** ^1^ Laboratory of Translational Oncology Intercollegiate Faculty of Biotechnology of the University of Gdańsk and the Medical University of Gdańsk Poland; ^2^ Centre of Biostatistics and Bioinformatics Medical University of Gdańsk Poland; ^3^ Department of Algorithms and Systems Modelling, Faculty of Electronics, Telecommunications and Informatics Gdańsk University of Technology Poland; ^4^ Department of Multimedia Systems, Faculty of Electronics, Telecommunications and Informatics Gdańsk University of Technology Poland; ^5^ Ideas, NCBR Warsaw Poland; ^6^ Molecular Medicine Program University of Utah Salt Lake City UT USA; ^7^ George E. Wahlen Veterans Affairs Medical Center Department of Internal Medicine and the Geriatric Research Education and Clinical Center (GRECC) Salt Lake City UT USA; ^8^ Department of Pathology University of Utah Salt Lake City UT USA; ^9^ Division of General Internal Medicine, Department of Internal Medicine University of Utah Salt Lake City UT USA

**Keywords:** gene expression, liquid biopsy, machine learning, platelets

## Abstract

Liquid biopsy demonstrates excellent potential in patient management by providing a minimally invasive and cost‐effective approach to detecting and monitoring cancer, even at its early stages. Due to the complexity of liquid biopsy data, machine‐learning techniques are increasingly gaining attention in sample analysis, especially for multidimensional data such as RNA expression profiles. Yet, there is no agreement in the community on which methods are the most effective or how to process the data. To circumvent this, we performed a large‐scale study using various machine‐learning techniques. First, we took a closer look at existing datasets and filtered out some patients to assert data collection quality. The final data collection included platelet RNA samples acquired from 1397 cancer patients (17 types of cancer) and 354 asymptomatic, presumed healthy, donors. Then, we assessed an array of different machine‐learning models and techniques (e.g., feature selection of RNA transcripts) in pan‐cancer detection and multiclass classification. Our results show that simple logistic regression performs the best, reaching a 68% cancer detection rate at a 99% specificity level, and multiclass classification accuracy of 79.38% when distinguishing between five cancer types. In summary, by revisiting classical machine‐learning models, we have exceeded the previously used method by 5% and 9.65% in cancer detection and multiclass classification, respectively. To ease further research, we open‐source our code and data processing pipelines (https://gitlab.com/jopekmaksym/improving‐platelet‐rna‐based‐diagnostics), which we hope will serve the community as a strong baseline.

## Graphical abstract

Platelet biopsy demonstrates excellent potential in patient management by providing a minimally invasive and cost‐effective approach to detecting and monitoring cancer. We assessed four machine‐learning models for pan‐cancer detection and multiclass classification. Our results show that the simplest model, logistic regression, outperforms other algorithms, reaching a 68% cancer detection at a 99% specificity level, and multiclass classification accuracy of 79.38%.

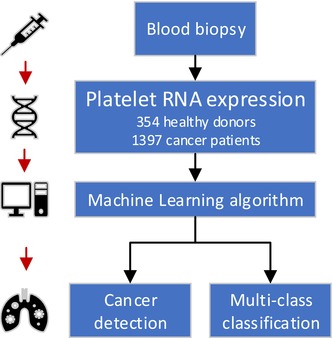

**Abbreviations**
AC: asymptomatic controls
AUC: area under curve
BalancedRF: balanced random forest
BRCA: breast cancer
cfDNA: circulating free DNA
cfRNA: circulating free RNA
CHOL: cholangiocarcinoma
CRC: colorectal cancer
CTCs: circulating tumor cells
DL: deep learning
ENDO: endometrial cancer
ESO: esophageal cancer
GLIO: glioma
HCC: hepatocellular carcinoma
HNSCC: head and neck squamous cell carcinoma
LogReg: logistic regression
LYM: lymphoma
MELA: melanoma
ML: machine learning
MM: multiple myeloma
NSCLC: non‐small cell lung cancer
OVCAR: ovarian cancer
PDAC: pancreatic ductal adenocarcinoma
PRCA: prostate cancer
PSO‐SVM: particle swarm optimized support vector machine
RCC: renal cell carcinoma
RF: random forest
ROC: receiver operating characteristics
SVM: support vector machine
URO: urothelial cancer

## 1 Introduction

Cancer is a devastating illness that claims millions of lives worldwide every year. In 2020 alone, it is estimated that approximately 10 million deaths were attributed to cancer [1]. There are numerous solutions for cancer diagnosis, but many techniques have certain limitations, such as invasiveness and high cost, or are not available in less developed parts of the world [1]. Furthermore, despite being highly specific, some methods may lack sensitivity in the early stages of the disease. Early detection of cancer is crucial for increasing the likelihood of successful treatment and survival [2]. As a result, it is essential to develop accurate and easily accessible diagnostic tools that are minimally invasive, especially for detecting early‐stage cancer.

Liquid biopsy has the potential to revolutionize cancer diagnosis as a less invasive and innovative approach [3, 4, 5, 6, 7, 8]. This technique provides valuable insights into tumors' genetic makeup by evaluating circulating biomarkers (i.e. circulating free DNA, cfDNA; circulating free RNA, cfRNA; extracellular vesicles; and circulating tumor cells, CTCs) predominantly in blood, with urine proving valuable for urothelial cancers [9, 10, 11, 12, 13, 14, 15, 16, 17, 18, 19, 20, 21]. In this manuscript, we focus on blood platelet RNA profiling as even aberrant platelet counts in morphology can alone serve as a marker of diseases such as bladder carcinoma or prostate cancer [22, 23, 24, 25]. The material especially valuable in this aspect is platelet and cancer detection is their RNA content which mirrors the host's response to the disease [26]. Platelets modify their RNA repertoire as a result of local and systemic signals. They play a crucial role in various biological processes such as inflammation, cancer progression, and metastasis, making their RNA signatures effective for cancer detection [27, 28]. By utilizing platelet RNA for detecting early‐stage cancer, clinicians can initiate timely interventions, thereby improving therapeutic efficacy and patient prognosis, especially when cancer detection is challenging. However, identifying the presence of cancer alone may not suffice for therapeutic decision‐making [29].

Dealing with highly dimensional RNA profiles is a complex task requiring robust computational techniques. Typically, this task involves working with thousands of features obtained from a relatively small number of samples. That is why many recent studies are using machine learning (ML) and deep learning (DL) to process and analyze samples to improve the accuracy of diagnosis [30]. Using advanced methods to tackle this problem does not always provide better outcomes, as simpler models are easier to interpret and thus provide some extent of explainability, making the method more reliable [31]. Employing the support vector machine (SVM) algorithm to analyze platelet RNA samples has already demonstrated remarkable success, starting a new era of non‐invasive cancer detection [15]. Advanced techniques such as the DL‐based imPlatelet classifier merge image‐based deep learning with transcriptomics to improve diagnostic precision [12]. In [13], a comprehensive analysis of platelet RNA using large‐scale datasets and advanced computational techniques such as convolutional neural networks and boosting achieved remarkable diagnostic accuracy in detecting cancer [14] utilized comparable CNN methods on platelet RNA data in the case of a multiclass classification task of six types of cancer. In [11], the authors developed RNA‐based blood tests using a particle swarm optimized support vector machine (PSO‐SVM) that could detect up to 18 cancer types by leveraging the unique properties of platelet RNA. This method identified cancer samples from asymptomatic individuals with 63% specificity at 99% specificity and accurately pinpointed the origin site of tumors for five different types of cancer with 68% accuracy. This research motivated us to apply our expertise in ML methods to enhance the diagnostic technique that uses platelet RNA for identifying cancer types.

This manuscript utilizes platelet RNA data collected from 354 healthy donors and 1397 cancer patients supplied in [11] and aims to enhance machine learning models for improved diagnostic accuracy. In this study, we:

- compare the performance of various machine learning models in differentiation between healthy and cancerous samples,
- compare the performance of various machine learning models in the multiclass,
- classification of five types of cancer, and
- investigate the impact of RNA transcript feature reduction on model performance.

## 2 Materials and methods

### 2.1 Dataset and sample processing

This investigation is based on publicly available raw platelet RNA samples from In 't Veld et al. [11]. Samples were collected and processed according to the guidelines established by Best et al. [17]. The dataset contained samples collected from January 2013 to June 2021 by 11 institutes from presumed healthy, asymptomatic controls (AC) and 17 types of cancer: breast cancer (BRCA), cholangiocarcinoma (CHOL), colorectal cancer (CRC), endometrial cancer (ENDO), esophageal cancer (ESO), glioma (GLIO), hepatocellular carcinoma (HCC), head and neck squamous cell carcinoma (HNSCC), lymphoma (LYM), melanoma (MELA), multiple myeloma (MM), non‐small cell lung cancer (NSCLC), ovarian cancer (OVCAR), pancreatic ductal adenocarcinomas (PDAC), prostate cancer (PRCA), renal cell carcinoma (RCC), and urothelial carcinoma (URO). Samples of ENDO and OVCAR patients were collected at the Department of Gynecology, Gynecological Oncology, and Gynecological Endocrinology at the Medical University of Gdansk (MUG). The study was approved by the Independent Ethics Committee of the Medical University of Gdansk (NKBBN/434/2017). All patients from all included hospitals signed informed consent forms. Procedures involving human subjects were in accordance with the Helsinki Declaration, as revised in 1983. All publicly available data was anonymized. An encompassing overview of the dataset's composition is provided in Table 1.

**Table 1:** Data overview. The aggregate count of samples included in the study are divided into the training, validation, and test sets and utilized during the machine learning process.

Cancer type	Data split [*n*]			
	Train	Validation	Test	Total
Asymptomatically healthy controls	104	104	146	354
Breast cancer	20	20	53	93
Cholangiocarcinoma	20	19	46	85
Colorectal cancer	19	19	46	84
Endometrial cancer	14	13	12	39
Esophageal cancer	0	0	15	15
Glioma	30	29	73	132
Hepatocellular carcinoma	7	8	8	23
Head and neck squamous cell carcinoma	20	20	61	101
Lymphoma	0	0	20	20
Melanoma	20	20	28	68
Multiple myeloma	10	10	11	31
Non‐small cell lung cancer	6	8	341	355
Ovarian cancer	16	17	102	135
Pancreatic ductal adenocarcinomas	20	20	86	126
Prostate cancer	12	11	11	34
Renal cell carcinoma	10	9	9	28
Urothelial carcinoma	10	9	9	28
Total	338	338	1077	1751

Based on the methodology described in Pastuszak et al. [12], we employed the DESeq2 package in R [32] for normalizing expression data through the variance stabilizing transformation [33]. The human reference genome (hg19) served as an annotative reference point in this process. To maintain the integrity and quality of our dataset, we omitted samples with fewer than 100 k total reads and solely incorporated genes backed by confirmed Gencode status. All the samples underwent uniform pre‐processing and were normalized collectively. Expression analysis of available samples highlighted significant differences between samples from the Netherlands Cancer Institute (NKI) and the remaining part of the cohort. These samples consisted of healthy donors (36), sarcoma (51), and former sarcoma (26) patients, NSCLC patients (167), ovarian cancer patients (9), and CRC and prostate cancer patients. Since sarcoma patients were underrepresented in other involved locations (2 sarcoma patients and no former sarcoma patients from outside NKI), further analysis was focused on healthy donors and NSCLC patients. After applying Benjamini–Hochberg FDR correction, 4916 transcripts out of 5346 considered were differentially expressed between NKI healthy donors and healthy donors from other locations. In the next step, a logistic regression classifier was trained to distinguish NKI samples from non‐NKI ones. 60% of healthy donors were assigned to the training set. Proportions between NKI and non‐NKI donors were preserved. The classifier was then tested on the remaining 40% of the healthy donors. Despite the class imbalance, the classifier managed to reach perfect classification with 100% accuracy and 100% ROC AUC. Feature importance analysis was performed, and features with the highest weights were selected for the pathway analysis. None of the pathways were significantly enriched after applying FDR correction. The same process was repeated for the NSCLC patients. After applying Benjamini–Hochberg FDR correction, 4626 transcripts out of 5346 considered were differentially expressed between NKI NSLC patients and NSCLC patients from other locations. The classifier managed to reach 88% accuracy and 86% balanced accuracy with 91.6% ROC AUC. The confusion matrix is presented in Fig. S1. Additionally, gene ontology analysis of the most important features in the classifier revealed, that five most enriched pathways were related to hemostasis and coagulation. These results are shown in Fig. S2. To further highlight the batch effect present in NKI samples, a heatmap was prepared (Fig. S3) showing that NKI samples tended to cluster together regardless of the patient group (NSCLC vs. HC). Since the contamination was suspected, the expression of hemoglobin was compared between NKI and non‐NKI samples. The observed hemoglobin expression levels were significantly higher in NKI samples. Results are presented in Fig. S4. This investigation showed that NKI samples might have been exposed to technical processing errors during the collection process, which has likely caused severe hemolysis and platelet activation. We excluded all NKI samples from the final dataset. Eventually, this bioinformatic processing yielded a data table of 1751 samples with 5349 transcript features each.

### 2.2 Sample classification

Our study focused on determining the optimal cancer diagnosis model for multiclass classification scenarios. We have compared multiple machine learning algorithms, such as logistic regression (LogReg), random forest (RF), balanced random forest (BalancedRF), and XGBOOST, and juxtaposed them with the original PSO‐SVM study [11]. Firstly, we tackled the pan‐cancer diagnosis task, differentiating between healthy controls and samples from all available cancer types grouped under one label, ‘Cancer’. In total, 2180 models were trained using train‐validation‐test data split from the original study, configured to a specificity threshold exceeding 99% to account for the larger proportion of healthy individuals within the population and ensure effective cancer identification for a future early cancer screening tool.

Secondly, we evaluated the performance of these algorithms for a multiclass classification task, identifying the tissue‐of‐origin of five cancer types with a minimal number of samples above one hundred to maintain a reliable sample size for training and testing purposes: GLIO, HNSCC, NSCLC, OVCAR, and PDAC. We employed a fivefold stratified cross‐validation [34] approach, training the models on all available transcript data as features, the 500 most variable features, and 500 features that scored the highest importance for each model on their first training. We reserved 20% of the data for testing and trained the models using 80% of the data (sampling based on various random seeds).

We leveraged *python* (version = 3.10) packages such as *scikit‐learn* (1.2.0), *xgboost* (1.7.6), *imblearn* (0.11.0), *numpy* (1.22.4), and *pandas* (1.2.4) to construct our machine learning models and software. We fine‐tuned the models' hyperparameters with GridSearchCV and provided a comprehensive list of parameters in the Table S2. Our testing metrics comprised specificity, sensitivity, accuracy (based on the initial prediction, and for the multiclass model, based on the second‐best prediction as well), balanced accuracy, f1 metric, and AUC (Area Under Curve) ROC (receiver operating characteristic). The bootstrap method was used to compute 95% CI (Confidence Interval). Code, package versions, and models are available at https://gitlab.com/jopekmaksym/improving‐platelet‐rna‐based‐diagnostics.

## 3 Results

### 3.1 Binary classification: pan‐cancer algorithm

**Fig. 1:**
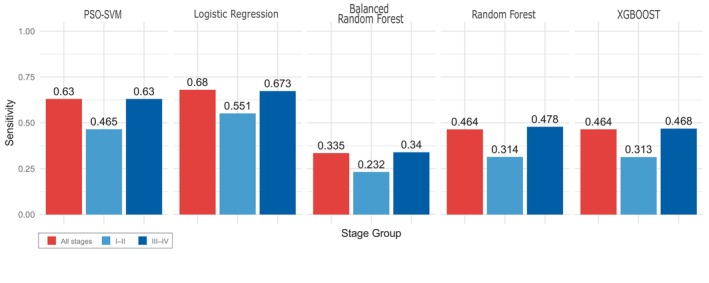
Performance comparison of models across test samples grouped into sets of all samples, early‐stage samples (stage I–II), and late stages (III–IV). PSO‐SVM (particle swarm optimized support vector machine) model metrics from the original study were added for comparison.

First, we trained and tested all binary classification models on the same train‐validation‐test data split used in In 't Veld et al. [11] (with and without the excluded samples). Next, we compared their performance against the previously proposed PSO‐SVM model. When comparing LogReg, RF, BalancedRF and XGBOOST methods, our investigation revealed that LogReg (hyperparameters: regularization = 0.1, penalty = l2, solver = newton‐cg, class‐weights = balanced) was the top‐performing model in terms of sensitivity at 99% specificity, achieving an impressive detection rate of 68% (64% after the inclusion of the excluded samples), as opposed to the 63% reached by In 't Veld et al. [11]. In all the performed experiments, XGBOOST was usually the second most sensitive model, reaching a higher score than LogReg in a few cases, but this algorithm was also the most dependable on random seed (the highest standard deviation between results). The performance of the logistic regression model compared to other tested machine learning algorithms is shown in Fig. 1.

**Fig. 2:**
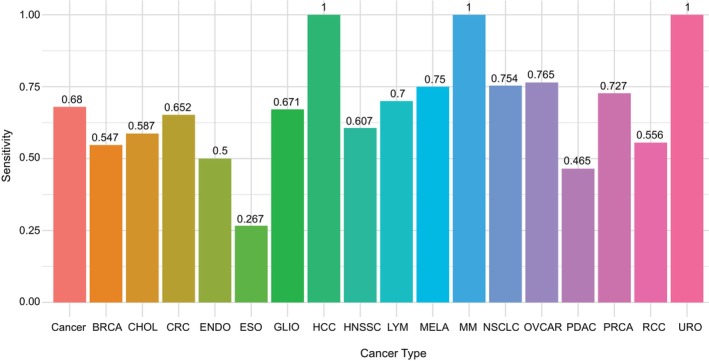
Sensitivity of the logistic regression model. The class ‘Cancer’ shows the overall performance of the classifier in detecting cancer (binary classification: ‘Healthy’ vs. ‘Cancer’). The remaining bars show the performance of the classifier across each cancer type separately. BRCA, breast cancer; CHOL, cholangiocarcinoma; CRC, colorectal cancer; ENDO, endometrial cancer; ESO, esophageal cancer; GLIO, glioma; HCC, hepatocellular carcinoma; HNSCC, head and neck squamous cell carcinoma; LYM, lymphoma; MELA, melanoma; MM, multiple myeloma; NSCLC, non‐small cell lung cancer; OVCAR, ovarian cancer; PDAC, pancreatic ductal adenocarcinoma; PRCA, prostate cancer; RCC, renal cell carcinoma; URO, urothelial cancer.

Beyond achieving remarkable performance, the LogReg model underscores the critical aspect of the interpretability of the classifier's predictions. By examining the significance of coefficients, LogReg facilitates the evaluation of the relative importance of various predictor variables (transcripts) in delineating the outcome, whether denoting ‘cancer’ or ‘healthy’. This affords a nuanced understanding of the particular RNAs that exert the most significant influence on the model's predictive capacity. The exploration of the feature importance of the cancer detection model has pinpointed the top five transcripts characterized by their largest weight on prediction outcomes: FKBP5, TMSB4XP8, MTRNR2L12, HBB, and SPDYC. Notably, LogReg demonstrated unparalleled performance in detecting BRCA, CHOL, CRC, GLIO, ENDO, HNSSC, MELA, OVCAR, and PDAC. However, it underperformed PSO‐SVM in detecting LYM and PRCA. These findings are visualized in Fig. 2.

**Fig. 3:**
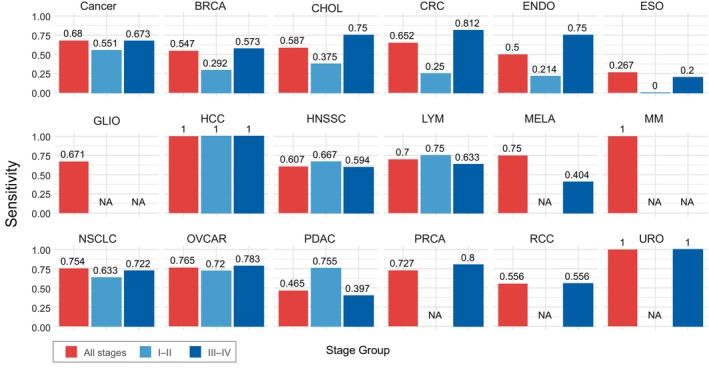
The sensitivity of the logistic regression model across all the cancer types and stages grouped into early stages (stage I–II) and late stages (III–IV). Cancer types that lack the data for specific stages are tagged as ‘NA’. The class ‘Cancer’ shows the overall performance across all the cancer types (binary classification ‘Healthy’ vs. ‘Cancer’). BRCA, breast cancer; CHOL, cholangiocarcinoma; CRC, colorectal cancer; ENDO, endometrial cancer; ESO, esophageal cancer; GLIO, glioma; HCC, hepatocellular carcinoma; HNSCC, head and neck squamous cell carcinoma; LYM, lymphoma; MELA, melanoma; MM, multiple myeloma; NSCLC, non‐small cell lung cancer; OVCAR, ovarian cancer; PDAC, pancreatic ductal adenocarcinoma; PRCA, prostate cancer; RCC, renal cell carcinoma; URO, urothelial cancer.

Cancer stage‐based analysis of the trained models showed that detecting cancer in its early stages still remains more challenging than detecting it in more advanced stages. Nevertheless, here, the LogReg model emerged as an especially effective method for detecting PDAC and OVCAR types of cancer, outperforming other methods by more than 20%. This is especially valuable given the poor outcomes of these types of cancer, with possible application for a screening test. Regrettably, none of the classifiers (including PSO‐SVM) could detect early‐stage esophageal (ESO) cancer. A comprehensive visual representation of stage‐based cancer detection using the LogReg model is available in Fig. 3.

Detailed performance comparison for pan‐cancer models' with respect to each stage is shown in Fig. S5 and stage‐based comparison is shown in Fig. S6, along with the detailed results for the most sensitive model in Table S3.

### 3.2 Multi‐class cancer sample classification

**Fig. 4:**
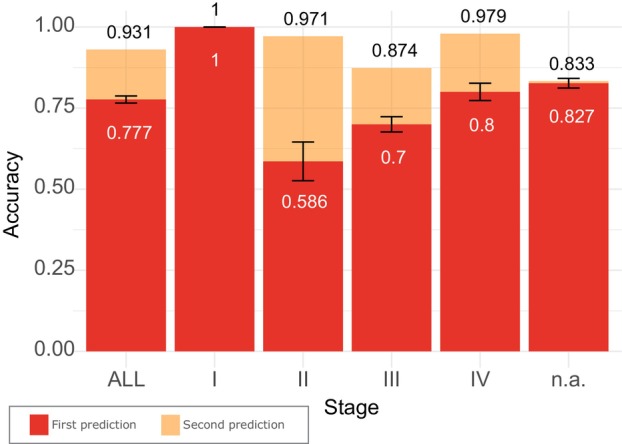
Detection accuracy for the first prediction and the second prediction on the test samples across all the cancer stages for multiclass models, based on five types of cancer. Error bars refer to the standard deviation acquired from all folds.

**Fig. 5:**
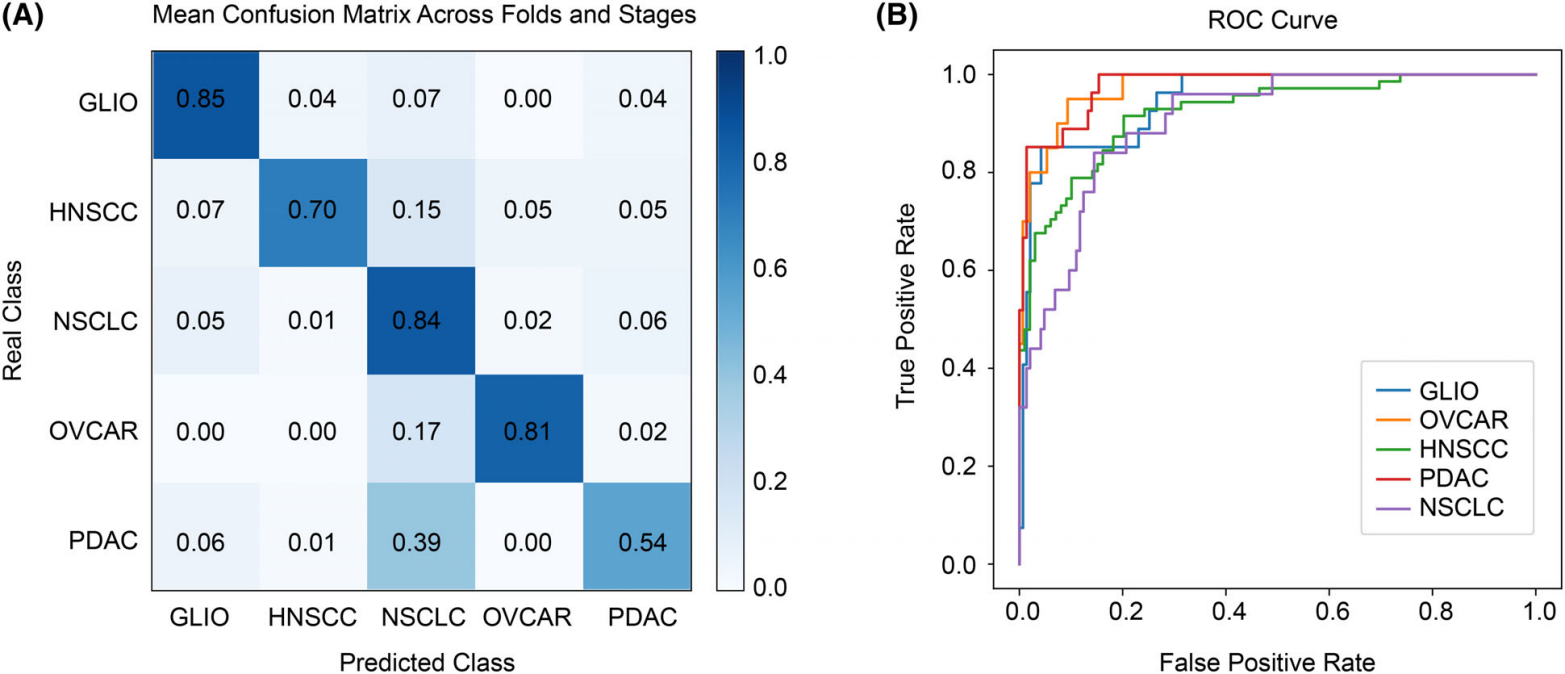
Classification results of the test samples for the most accurate model. (A) Mean, normalized confusion matrix across all the folds and stages. (B) ROC curve for each analyzed cancer type. GLIO, glioma; HNSCC, head and neck squamous cell carcinoma; NSCLC, non‐small cell lung cancer; OVCAR, ovarian cancer; PDAC, pancreatic ductal adenocarcinoma.

Our next step was to find the most accurate model in the multiclass classification of samples from five types of cancer: GLIO, HNSCC, NSCLC, OVCAR, and PDAC. Comparing the performance of all tested algorithms, The LogReg algorithm once again proved to be the most effective approach. It achieved an impressive 77.65% mean accuracy across all folds in the first prediction, whereas the previously proposed method, PSO‐SVM, reached 68%. In the second‐best prediction, the LogReg model achieved an accuracy of 93.06%, while the PSO‐SVM model reached 85%. Compared to other tested models, LogReg exhibited the lowest standard deviation across the folds and random seeds compared to other models' performance, further indicating its robustness. Upon analyzing the accuracy of multiclass classification with respect to the stage of cancer, the results revealed that stage II cancer was the most challenging to classify and displayed the highest standard deviation among folds. Surprisingly, stage I cancer was the easiest to classify, scoring 99.9% accuracy in each fold. These findings are further visualized in Fig. 4. Detailed per‐stage performance of the most accurate model is shown in Table 2. As for the classification of specific cancer types, GLIO scored the highest overall accuracy of 85%. The second most correctly classified was NSCLC, but this cancer type also had the highest false discovery rate. A comprehensive confusion matrix is shown along the ROC curves in Fig. 5.

**Table 2:** Performance of the most accurate model (LogReg).

Stages	Accuracy [%]
All stages	79.38
Stage I	99.99
Stage II	50.00
Stage III	73.68
Stage IV	86.62
Unknown stage	80.00

Performance comparison for all tested multiclass models' is shown as Figs S7 and S8.

### 3.3 Impact of feature reduction

During our work with cancer detection algorithms and feature reduction, we have discovered that reducing the number of features can boost the models' performance in some cases. Re‐training the models on a subset of features that showed the highest impact on the classifier decision usually improved the accuracy by approximately 10%. The implementation of this approach yielded the highest performance improvements for the random forest and balanced random forest models. However, despite this advantage, their predictive scores remained inferior to those of the other models, namely LogReg, XGBOOST, and PSO‐SVM. The only model that did not take advantage of the implementation of feature reduction was LogReg. Contrary to these findings, it was observed that in the case of multiclass classification, the reduction in feature count did not exhibit a notable impact on the efficacy of the models. In the majority of instances, the accuracy only displayed negligible variability of less than 10%, depending on the feature set. A detailed comparison is presented in Figs S9 and S10.

## 4 Discussion

The realm of cancer diagnosis is constantly evolving, with liquid biopsy emerging as an increasingly valuable asset. As platelets possess distinctive RNA profiles, measuring the host response to disease shows promise in patient management. The pathological alterations that mirror an individual's response to illness can be leveraged by integrating the computational capabilities of machine learning [16].

Our study delved into the diagnostic efficiency of various machine learning models, yielding enlightening findings. In the case of cancer detection, logistic regression scored 68% sensitivity at 99% specificity threshold on the test set of 931 cancer samples belonging to 17 different types and 146 healthy donor samples, consistently outperforming all other tested models and the PSO‐SVM model previously proposed by [11]. According to our knowledge, this is the highest detection accuracy result obtained for platelet RNA studies on such a large set of data. This suggests that while advanced algorithms such as PSO‐SVM offer reasonable accuracy, traditional models such as logistic regression can sometimes deliver superior results, depending on the data's context and characteristics as well as on the process of hyperparameter optimization. Moreover, our methods achieved comparable results to the model performance described in Cygert et al., and the Galleri® test from Grail (branded as a breakthrough device by FDA) which is based on cfDNA circulating in blood [5, 13]. While Cygert et al. [13] obtained slightly higher accuracy (89%), our models' performance can be considered more robust because of the greater number of samples (343 platelet RNA samples in the described article) and cancer types (17, as opposed to 6 in Cygert et al. [13]). Furthermore, we have obtained significantly higher performance in the case of healthy donor classification. In contrast, the Galleri test used by [5] scored only 55% sensitivity at 99% specificity level, despite the fact that there were more samples used, namely 1422 cancer cases belonging to above 20 different types and 879 healthy donors. The recent Pathfinder study aimed to evaluate methylated cfDNA of cancer patients further, providing new insights on test applications in real‐world data [35]. Another promising, clinically available diagnostic test is CancerSEEK reported by Cohen et al. [6]. The latter platform would combine protein biomarker concentrations and mutations in cfDNA, reaching a remarkable 70% sensitivity at a 98% specificity level. However, this test proved limited applicability for the detection of early stages of cancer, resulting in only 40% sensitivity for stage I patients.

As far as multiclass cancer classification is concerned, our methods achieved remarkable results, namely 77.65% accuracy in top‐1 prediction and 93.06% in top‐2 prediction, classifying 806 samples into five types of cancer. However, we have observed a sudden drop in models' performance regarding the classification of stage II cancer. While the platelet transcriptome in healthy donors remains relatively stable, as we have proven before, what we believe happens is that the RNA profile changes very dynamically in response to disease, even in the early stages [36]. The platelet pattern is indicative of the current disease localization. As cancer progresses and spreads, the clarity of the platelet RNA profile diminishes, resulting in a decrease in accuracy. Furthermore, cancer‐specific features in transcriptomic platelet profiles of patients with metastatic disease may be obscured by signals coming from multiple tumor sites and the systemic nature of the advanced disease likely introduces additional noise. To our best knowledge, this model performance surpasses all previous results from the current literature. Namely in [15] for the classification of six types of cancer based on 283 samples and 55 healthy donors, top‐1 accuracy was equal to 71% and top‐2 accuracy: 89%. Moreover, recent methods from [11] are also characterized by lower performance than our approach. Based on the same dataset and the same types of cancers as in this study, the top‐1 accuracy was equal to 68% and top‐2: 85%. This concludes that our proposed methods achieve the highest performance on a reliable number of samples.

Addressing the challenge of detecting cancer in its early stages was evident across all our developed models. Early detection is crucial for successful cancer treatment; therefore, the models' limitations in identifying cancers such as HNSSC and ESO in their early stages highlight an area that requires further investigation. However, the effectiveness of logistic regression in detecting PDAC and OVCAR cancers, which is critical for the early diagnosis of these cancer types, presents an opportunity for future screening tests. Logistic regression and PSO‐SVM models demonstrated high performance on different cancer types, indicating that they could be used interchangeably to improve overall cancer detection, depending on the state of the patient's health.

A deeper examination of the feature importance of the cancer detection model has revealed the top five transcripts at play: *FKBP5*, *TMSB4XP8*, *MTRNR2L12*, *HBB*, and *SPDYC*. All of these genes were upregulated in cancer patients. Among them, the *FKBP5* is reported to be overexpressed in the primary tumors of brain cancer, prostate cancer, lymphoma, head and neck cancer, colorectal cancer, and downregulated in pancreatic cancer [37, 38, 39]. *FKBP5* is a negative regulator of the AKT pathway, with potential implications for response to chemotherapy [39]. Furthermore, *MTRNR2L12* pseudo‐gene, *HBB*, and *SPDYC* were already reported as possible prognostic markers in breast cancer, where their overexpression in tumor cells led to unfavorable prognosis [40, 41, 42, 43]. The *HBB* gene was also proposed as a possible biomarker by Kurota et al. [44] *HBB*‐positive renal cell carcinoma patients had a higher recurrence rate and shorter survival than HBB‐negative patients. Importantly, our investigation revealed a novel cancer disease indicator, *TMSB4XP8*, potentially stemming from off‐target overexpression. This pseudogene has no previous evidence of its relation to cancer in the literature, but is related to *TMSB4X*, which is associated with tumor progression, and metastasis, and was proposed as an NSCLC prognostic biomarker by Yang et al. [45]. Even though pseudogenes are not directly implicated in cancer progression, they may become transcriptionally active due to alterations in regulatory elements of chromatin structure [46]. Although biologically irrelevant, their deregulation might be indicative of cancer and thus be of diagnostic significance.

The technique of feature reduction allowed us to boost the sensitivity of most of our models, possibly filtering the unnecessary noise. This change improved the performance by around 10%, which was especially observable among the tree‐based models as their performance was the lowest. Unfortunately, our methods of the implementation of feature reduction did not improve our most sensitive algorithm (LogReg). While the change in model performance was usually not significant, this approach effectively accelerated the algorithm training process. Alternative feature reduction methods may be better suited for this task.

Although our research provides valuable insights further improving the current state of early cancer detection methods, there are some limitations and future works that need to be further investigated. Our first concern is data availability. While ML methods require a huge amount of data, there are not many available datasets for working with platelet RNA data, and some of them are provided with limited access [47]. There is also a need for a well‐described benchmark dataset for algorithm performance comparison as it is commonly used in other ML appliances [48, 49]. This way even more optimized algorithm could be found. We also want to acknowledge that while blood remains one of the most consistently reliable sources for liquid biopsy‐based cancer detection, it may not always be the optimal choice for certain cancer types. For example, in urological cancers, urine emerges as a valuable alternative, offering useful insights at even lesser invasiveness [18, 50, 51, 52, 53, 54]. To serve the community, we open‐source our code with the hope that it can serve as a strong baseline. In addition, we think that there is still an open ground for the exploration of feature reduction methods that could not only enhance the performance of models but also optimize the sample collection process. Extracting features based on biological or therapeutic insights would be a valuable next step in developing a clinic‐ready cancer detection toolkit.

## 5 Conclusions

Our research underscores the significant potential of liquid biopsy and platelet RNA profiles in the early detection and classification of various cancer types. By leveraging machine learning models, we have demonstrated that traditional algorithms such as logistic regression can achieve superior diagnostic performance compared to more complex models under certain conditions. Our findings highlight the importance of model selection and hyperparameter optimization in enhancing diagnostic accuracy. Logistic regression emerged as the most effective model, achieving a sensitivity of 68% at a 99% specificity threshold, and 77.65% balanced accuracy in multiclass cancer prediction of GLIO, HNSCC, NSCLC, OVCAR, and PDAC, which is a notable improvement over previously proposed methods. This suggests that simpler models, when properly optimized, can sometimes outperform advanced algorithms in specific applications. The identification of key transcripts used in model decision‐making, such as *FKBP5*, *TMSB4XP8*, *MTRNR2L12*, *HBB*, and *SPDYC* in our case, offers promising biomarkers for future diagnostic applications. The novel discovery of *TMSB4XP8* as a potential cancer indicator warrants further investigation. Our approach to feature reduction boosted the cancer detection performance of all models except our most sensitive algorithm (LogReg). Other methods might provide better results. Our research provides a strong foundation for the development of more accurate and robust liquid biopsy‐based cancer detection tools. By open‐sourcing our code, we aim to facilitate further advancements in this field, ultimately contributing to earlier and more reliable cancer diagnosis, which is crucial for improving patient outcomes.

## Conflict of interest

The authors declare no conflict of interest.

## Author contributions

MAJ, AS, and KP conceptualized and designed the study. MAJ and KP performed data processing. MAJ, MS, and KP performed the experiments and analysis. MAJ, AS, MS, KP, MTR, SC, and AJŻ interpreted the results. MAJ and MS visualized the data. MAJ, AS, MS, KP, and SC wrote the draft of the manuscript. AS and AJŻ supervised the study and provided funding.

### Peer review

The peer review history for this article is available at https://www.webofscience.com/api/gateway/wos/peer‐review/10.1002/1878‐0261.13689.

## Supporting information


**Fig. S1.** Confusion matrix for classification of NKI vs. non‐NKI NSCLC patients.
**Fig. S2.** Gene ontology analysis of the most important features for model distinguishing NKI vs. non‐NKI NSCL patients.
**Fig. S3.** Expression heatmap showing healthy controls and NSCLC patients.
**Fig. S4.** Comparison of hemoglobin expression levels in asymptomatically healthy controls and non‐small cell lung cancer based on site of sample collection.
**Fig. S5.** Sensitivity of models based on the cancer type.
**Fig. S6.** Sensitivity of models based on cancer type and stage.
**Fig. S7.** Detection accuracy for first prediction (dark color) and second prediction (light color) on test samples across various random seeds for multiclass models based on 5 types of cancer.
**Fig. S8.** Detection accuracy for first prediction (dark color) and second prediction (light color) on test samples across all cancer stages for multiclass models based on 5 types of cancer.
**Fig. S9.** Sensitivity of models based on cancer type and feature group.
**Fig. S10.** Detection accuracy for the first prediction across various random states for multiclass models based on 5 types of cancer.
**Table S1.** Detailed amount of samples available from each stage of cancer.
**Table S2.** Grid search parameters used for model hyperparameter optimization.
**Table S3.** Pan‐cancer classification metrics for the most sensitive model.

## Data Availability

The code, sample metadata along with train‐validation‐test split information, and sample data are available in GitLab, at https://gitlab.com/jopekmaksym/improving‐platelet‐rna‐based‐diagnostics. The datasets were derived from sources in the public domain: In 't Veld et al. [11].

## References

[1] Sung H , Ferlay J , Siegel RL , Laversanne M , Soerjomataram I , Jemal A , et al. Global cancer statistics 2020: GLOBOCAN estimates of incidence and mortality worldwide for 36 cancers in 185 countries. CA Cancer J Clin. 2021;71:209–249. 10.3322/caac.21660 33538338

[2] Rowlands S . Cancer survival by stage at diagnosis for England (experimental statistics) – Office for National Statistics — ons.gov.uk. [Accessed 24th October 2023].

[3] Alix‐Panabières C , Pantel K . Liquid biopsy: from discovery to clinical application. Cancer Discov. 2021;11:858–873. 10.1158/2159-8290.CD-20-1311 33811121

[4] Lamb YN , Dhillon S . Epi proColon® 2.0 CE: a blood‐based screening test for colorectal cancer. Mol Diagn Ther. 2017;21:225–232. 10.1007/s40291-017-0259-y 28155091

[5] GRAIL announces positive new data with multi‐cancer early detection blood test from CCGA study – GRAIL. Available from: grail.com.

[6] Cohen JD , Li L , Wang Y , Thoburn C , Afsari B , Danilova L , et al. Detection and localization of surgically resectable cancers with a multi‐analyte blood test. Science. 2018;359:926–930. 10.1126/science.aar3247 29348365 PMC6080308

[7] Safrastyan A , Wollny D . Network analysis of hepatocellular carcinoma liquid biopsies augmented by single‐cell sequencing data. Front Genet. 2022;13:921195. 10.3389/fgene.2022.921195 36092896 PMC9452847

[8] Heidrich I , Deitert B , Werner S , Pantel K . Liquid biopsy for monitoring of tumor dormancy and early detection of disease recurrence in solid tumors. Cancer Metastasis Rev. 2023;42:161–182. 10.1007/s10555-022-10075-x 36607507 PMC10014694

[9] Zhang Q , Song X , Song X . Contents in tumor‐educated platelets as the novel biosource for cancer diagnostics. Front Oncol. 2023;13:1165600. 10.3389/fonc.2023.1165600 37139159 PMC10151018

[10] Heinhuis KM , In 't Veld SGJG , Dwarshuis G , van den Broek D , Sol N , Best MG , et al. RNA‐sequencing of tumor‐educated platelets, a novel biomarker for blood‐based sarcoma diagnostics. Cancer. 2020;12:1372. 10.3390/cancers12061372 PMC7352477 32471035

[11] In 't Veld SGJG , Arkani M , Post E , Antunes‐Ferreira M , D'Ambrosi S , Vessies DCL , et al. Detection and localization of early‐ and late‐stage cancers using platelet RNA. Cancer Cell. 2022;40:999–1009.e6. 10.1016/j.ccell.2022.08.006 36055228

[12] Pastuszak K , Supernat A , Best MG , In 't Veld SGJG , Łapińska‐Szumczyk S , Łojkowska A , et al. imPlatelet classifier: image‐converted RNA biomarker profiles enable blood‐based cancer diagnostics. Mol Oncol. 2021;15:2688–2701. 10.1002/1878-0261.13014 34013585 PMC8486571

[13] Cygert S , Pastuszak K , Górski F , Sieczczyński M , Juszczyk P , Rutkowski A , et al. Platelet‐based liquid biopsies through the lens of machine learning. Cancer. 2023;15:2336. 10.3390/cancers15082336 PMC10136732 37190262

[14] Jopek MA , Pastuszak K , Cygert S , Best MG , Wurdinger T , Jassem J , et al. Deep learning‐based, multiclass approach to cancer classification on liquid biopsy data. IEEE J Transl Eng Health Med. 2024;12:306–313. 10.1109/jtehm.2024.3360865

[15] Best MG , Sol N , Kooi I , Tannous J , Westerman BA , Rustenburg F , et al. RNA‐seq of tumor‐educated platelets enables blood‐based pan‐cancer, multiclass, and molecular pathway cancer diagnostics. Cancer Cell. 2015;28:666–676. 10.1016/j.ccell.2015.09.018 26525104 PMC4644263

[16] Best MG , Sol N , In 't Veld SGJG , Vancura A , Muller M , Niemeijer A‐LN , et al. Swarm intelligence‐enhanced detection of non‐small‐cell lung cancer using tumor‐educated platelets. Cancer Cell. 2017;32:238–252.e9. 10.1016/j.ccell.2017.07.004 28810146 PMC6381325

[17] Best MG , In 't Veld SGJG , Sol N , Wurdinger T . RNA sequencing and swarm intelligence‐enhanced classification algorithm development for blood‐based disease diagnostics using spliced blood platelet RNA. Nat Protoc. 2019;14:1206–1234. 10.1038/s41596-019-0139-5 30894694

[18] Alberca‐del Arco F , Prieto‐Cuadra D , Santos‐Perez de la Blanca R , Sáez‐Barranquero F , Matas‐Rico E , Herrera‐Imbroda B . New perspectives on the role of liquid biopsy in bladder cancer: applicability to precision medicine. Cancer. 2024;16:803. 10.3390/cancers16040803 PMC10886494 38398192

[19] Pantel K . Liquid biopsy: blood‐based analyses of ctDNA and CTCs. Clin Chem. 2021;67:1437–1439. 10.1093/clinchem/hvab168 34549290

[20] Groen L , Schalken J . Liquid biopsy for prostate and bladder cancer: progress and pitfalls. Eur Urol Focus. 2022;8:904–906. 10.1016/j.euf.2022.08.013 36123280

[21] Cortés‐Hernández LE , Eslami‐S Z , Pantel K , Alix‐Panabières C . Circulating tumor cells: from basic to translational research. Clin Chem. 2024;70:81–89. 10.1093/clinchem/hvad142 38175586 PMC10765989

[22] Wang X , Cui M‐M , Xu Y , Liu L , Niu Y , Liu T , et al. Decreased mean platelet volume predicts poor prognosis in invasive bladder cancer. Oncotarget. 2017;8:68115–68122. 10.18632/oncotarget.19242 28978101 PMC5620241

[23] Deng D , Li X , Qi T , Dai Y , Liu N , Li H . A novel platelet risk score for stratifing the tumor immunophenotypes, treatment responses and prognosis in bladder carcinoma: results from real‐world cohorts. Front Pharmacol. 2023;14:1187700. 10.3389/fphar.2023.1187700 37214475 PMC10192868

[24] Mezei T , Bőde I , Tenke P , Jósa V , Merkel K , Szilasi Z , et al. The correlation between platelet count and survival in prostate cancer. Res Rep Urol. 2022;14:193–202. 10.2147/rru.s359715 35572814 PMC9092472

[25] Chen M , Hou L , Hu L , Tan C , Wang X , Bao P , et al. Platelet detection as a new liquid biopsy tool for human cancers. Front Oncol. 2022;12:983724. 10.3389/fonc.2022.983724 36185270 PMC9515491

[26] In 't Veld SGJG , Wurdinger T . Tumor‐educated platelets. Blood. 2019;133:2359–2364. 10.1182/blood-2018-12-852830 30833413

[27] Gay LJ , Felding‐Habermann B . Contribution of platelets to tumour metastasis. Nat Rev Cancer. 2011;11:123–134. 10.1038/nrc3004 21258396 PMC6894505

[28] Sol N , In 't Veld SGJG , Vancura A , Tjerkstra M , Leurs C , Rustenburg F , et al. Tumor‐educated platelet RNA for the detection and (pseudo)progression monitoring of glioblastoma. Cell Rep Med. 2020;1:100101. 10.1016/j.xcrm.2020.100101 33103128 PMC7576690

[29] Hoadley KA , Yau C , Hinoue T , Wolf DM , Lazar AJ , Drill E , et al. Cell‐of‐origin patterns dominate the molecular classification of 10, 000 tumors from 33 types of cancer. Cell. 2018;173:291–304.e6. 10.1016/j.cell.2018.03.022 29625048 PMC5957518

[30] Bostanci E , Kocak E , Unal M , Guzel MS , Acici K , Asuroglu T . Machine learning analysis of RNA‐seq data for diagnostic and prognostic prediction of colon cancer. Sensors. 2023;23:3080. 10.3390/s23063080 36991790 PMC10052105

[31] Lu S‐C , Swisher CL , Chung C , Jaffray D , Sidey‐Gibbons C . On the importance of interpretable machine learning predictions to inform clinical decision making in oncology. Front Oncol. 2023;13:1129380. 10.3389/fonc.2023.1129380 36925929 PMC10013157

[32] Love MI , Huber W , Anders S . Moderated estimation of fold change and dispersion for RNA‐seq data with DESeq2. Genome Biol. 2014;15:550. 10.1186/s13059-014-0550-8 25516281 PMC4302049

[33] Huber W , von Heydebreck A , Sültmann H , Poustka A , Vingron M . Variance stabilization applied to microarray data calibration and to the quantification of differential expression. Bioinformatics. 2002;18(Suppl 1):S96–S104. 10.1093/bioinformatics/18.suppl_1.s96 12169536

[34] Berrar D . Cross‐validation. In: Ranganathan S , Gribskov M , Nakai K , Schönbach C , editors. Encyclopedia of bioinformatics and computational biology. Amsterdam: Elsevier; 2019. p. 542–545.

[35] Schrag D , Beer TM , McDonnell CH , Nadauld L , Dilaveri CA , Reid R , et al. Blood‐based tests for multicancer early detection (PATHFINDER): a prospective cohort study. Lancet. 2023;402:1251–1260. 10.1016/S0140-6736(23)01700-2 37805216 PMC11027492

[36] Supernat A , Popęda M , Pastuszak K , Best MG , Grešner P , In 't Veld SGJG , et al. Transcriptomic landscape of blood platelets in healthy donors. Sci Rep. 2021;11:15679. 10.1038/s41598-021-94003-z 34344933 PMC8333095

[37] Li L , Lou Z , Wang L . The role of FKBP5 in cancer aetiology and chemoresistance. Br J Cancer. 2010;104:19–23. 10.1038/sj.bjc.6606014 21119664 PMC3039800

[38] Liu T , Wang C , Xia Z . Overexpressed FKBP5 mediates colorectal cancer progression and sensitivity to FK506 treatment via the NF‐κB signaling pathway. FEBS J. 2024. 10.1111/febs.17126 38602236

[39] Pei H , Li L , Fridley BL , Jenkins GD , Kalari KR , Lingle W , et al. FKBP51 affects cancer cell response to chemotherapy by negatively regulating Akt. Cancer Cell. 2009;16:259–266. 10.1016/j.ccr.2009.07.016 19732725 PMC2755578

[40] Ruan Y , Tang Q , Qiao J , Wang J , Li H , Yue X , et al. Identification of a novel glycolysis‐related prognosis risk signature in triple‐negative breast cancer. Front Oncol. 2023;13:1171496. 10.3389/fonc.2023.1171496 37274269 PMC10233057

[41] Ponzetti M , Capulli M , Angelucci A , Ventura L , Monache SD , Mercurio C , et al. Non‐conventional role of haemoglobin beta in breast malignancy. Br J Cancer. 2017;117:994–1006. 10.1038/bjc.2017.247 28772282 PMC5625664

[42] Wang F , Tang C , Gao X , Xu J . Identification of a six‐gene signature associated with tumor mutation burden for predicting prognosis in patients with invasive breast carcinoma. Ann Transl Med. 2020;8:453. 10.21037/atm.2020.04.02 32395497 PMC7210212

[43] Alsaleem MA , Ball G , Toss MS , Raafat S , Aleskandarany M , Joseph C , et al. A novel prognostic two‐gene signature for triple negative breast cancer. Mod Pathol. 2020;33:2208–2220. 10.1038/s41379-020-0563-7 32404959

[44] Kurota Y , Takeda Y , Ichiyanagi O , Saitoh S , Ito H , Naito S , et al. Hemoglobin β expression is associated with poor prognosis in clear cell renal cell carcinoma. Biomedicine. 2023;11:1330. 10.3390/biomedicines11051330 PMC10216096 37239002

[45] Yang Z , Luo J , Zhang M , Zhan M , Bai Y , Yang Y , et al. TMSB4X: a novel prognostic marker for non‐small cell lung cancer. Heliyon. 2023;9:e21505. 10.1016/j.heliyon.2023.e21505 38027718 PMC10663839

[46] Nakamura‐García AK , Espinal‐Enríquez J . Pseudogenes in cancer: state of the art. Cancer. 2023;15:4024. 10.3390/cancers15164024 PMC10452131 37627052

[47] Gao Y , Liu C‐J , Li H‐Y , Xiong X‐M , Li G‐L , In 't Veld SGJG , et al. Platelet RNA enables accurate detection of ovarian cancer: an intercontinental, biomarker identification study. Protein Cell. 2022;14:579–590. 10.1093/procel/pwac056 PMC10246718 36905391

[48] Krizhevsky A , Hinton G . Learning multiple layers of features from tiny images. 2009.

[49] Johnson AEW , Pollard TJ , Shen L , Lehman LH , Feng M , Ghassemi M , et al. MIMIC‐III, a freely accessible critical care database. Sci Data. 2016;3:160035. 10.1038/sdata.2016.35 27219127 PMC4878278

[50] Oshi M , Murthy V , Takahashi H , Huyser M , Okano M , Tokumaru Y , et al. Urine as a source of liquid biopsy for cancer. Cancer. 2021;13:2652. 10.3390/cancers13112652 PMC8199052 34071230

[51] Satyal U , Srivastava A , Abbosh PH . Urine biopsy—liquid gold for molecular detection and surveillance of bladder cancer. Front Oncol. 2019;9:1266. 10.3389/fonc.2019.01266 31803629 PMC6877686

[52] Guo J , Zhang X , Xia T , Johnson H , Feng X , Simoulis A , et al. Non‐invasive urine test for molecular classification of clinical significance in newly diagnosed prostate cancer patients. Front Med. 2021;8:721554. 10.3389/fmed.2021.721554 PMC8476767 34595190

[53] Tosoian JJ , Zhang Y , Xiao L , Xie C , Samora NL , Niknafs YS , et al. Development and validation of an 18‐gene urine test for high‐grade prostate cancer. JAMA Oncol. 2024. 10.1001/jamaoncol.2024.0455 PMC11190811 38635241

[54] Yang M , Liu X , Tang X , Sun W , Ji Z . LC‐MS based urine untargeted metabolomic analyses to identify and subdivide urothelial cancer. Front Oncol. 2023;13:1160965. 10.3389/fonc.2023.1160965 37256175 PMC10226587

