# Supplementary material for: Improving platelet‐RNA‐based diagnostics: a comparative analysis of machine learning models for cancer detection and multiclass classification
Source: Mol Oncol. 2024 Jun 17;18(11):2743–54. doi: 10.1002/1878-0261.13689 (PMC11547247; doi:10.1002/1878-0261.13689)
Supplement: Supplementary file 1 — Fig. S1. Confusion matrix for classification of NKI vs. non‐NKI NSCLC patients. Fig. S2. Gene ontology analysis of the most important features for model distinguishing NKI vs. non‐NKI NSCL patients. Fig. S3. Expression heatmap showing healthy controls and NSCLC patients. Fig. S4. Comparison of hemoglobin expression levels in asymptomatically healthy controls and non‐small cell lung cancer based on site of sample collection. Fig. S5. Sensitivity of models based on the cancer type. Fig. S6. Sensitivity of models based on cancer type and stage. Fig. S7. Detection accuracy for first prediction (dark color) and second prediction (light color) on test samples across various random seeds for multiclass models based on 5 types of cancer. Fig. S8. Detection accuracy for first prediction (dark color) and second prediction (light color) on test samples across all cancer stages for multiclass models based on 5 types of cancer. Fig. S9. Sensitivity of models based on cancer type and feature group. Fig. S10. Detection accuracy for the first prediction across various random states for multiclass models based on 5 types of cancer. Table S1. Detailed amount of samples available from each stage of cancer. Table S2. Grid search parameters used for model hyperparameter optimization. Table S3. Pan‐cancer classification metrics for the most sensitive model. [file MOL2-18-2743-s001.pdf]

Molecular Oncology | Supplementary Material

Improving platelet-RNA-based diagnostics: a comparative analysis of machine learning models for cancer detection and multi-class classification

Maksym A. Jopek[1,2], Krzysztof Pastuszak[1,2,3], Michał Sieczczyński[1,2], Sebastian Cygert[4-5], Anna J. Żaczek[1], Matthew T. Rondina[6-9], Anna Supernat[1,2]

[1]Laboratory of Translational Oncology, Intercollegiate Faculty of Biotechnology, University of Gdańsk and Medical University of Gdańsk, Gdańsk, Poland; [2]Centre of Biostatistics and Bioinformatics, Medical University of Gdańsk, Gdańsk, Poland [3]Department of Algorithms and Systems Modelling, Faculty of Electronics, Telecommunications and Informatics, Gdańsk University of Technology, Gdańsk, Poland; [4]Department of Multimedia Systems, Faculty of Electronics, Telecommunications, and Informatics, Gdańsk University of Technology, Gdańsk, Poland. [5]NCBR, Warsaw, Poland; [6]University of Utah Molecular Medicine Program, Salt Lake City, UT; [7]Department of Internal Medicine, Division of General Internal Medicine, University of Utah, Salt Lake City, UT; [8]George E. Wahlen Veterans Affairs Medical Center Department of Internal Medicine and the Geriatric Research Education and Clinical Center (GRECC), Salt Lake City, UT; [9]Department of Pathology, University of Utah, Salt Lake City, UT,

Abbreviations:
SVM - support vector machine,
PSO-SVM - particle swarm optimized support vector machine,
LogReg - logistic regression,
RF - random forest,
BalancedRF - balanced random forest,
AUC – area under curve,
ROC – receiver operating characteristics,
BRCA – breast cancer,
CHOL – cholangiocarcinoma,
CRC – colorectal cancer,
ENDO – endometrial cancer,
ESO – esophageal cancer,
GLIO – glioma,
HCC – hepatocellular carcinoma,
HNSCC – head and neck squamous cell carcinoma,
LYM – lymphoma,
MELA – melanoma,
MM – multiple myeloma,
NSCLC – non-small cell lung cancer,
OVCAR – ovarian cancer,
PDAC – pancreatic ductal adenocarcinoma,
PRCA – prostate cancer,
RCC – renal cell carcinoma,
URO – urothelial cancer.
NKI - Netherlands Cancer Institute

Supplemental Material | Tables

Table S1 Detailed amount of samples available from each stage of cancer.

Sample type	**Stage**				
	**I**	**II**	**III**	**IV**	**n.a.**
Healthy	-	-	-	-	354
Cancer	93	151	196	718	239
BRCA	12	18	4	57	2
CHOL	6	22	9	45	3
CRC	1	3	5	58	17
ENDO	24	4	8	0	3
ESO	0	1	10	1	3
GLIO	0	0	0	0	132
HCC	1	1	2	11	8
HNSSC	4	7	30	60	0
LYM	2	5	5	6	2
MELA	0	2	6	58	2
MM	0	0	0	0	31
NSCLC	10	6	36	293	10
OVCAR	30	16	48	35	6
PDAC	3	66	31	25	1
PRCA	0	0	0	15	19
RCC	0	0	0	28	0
URO	0	0	2	26	0

Table S2 Grid Search parameters used for model hyperparameter optimization.

**MODEL TYPE**	**GRID SEARCH PARAMETERS**
RANDOM FOREST,<br>BALANCED RANDOM FOREST	'n_estimators': [100, 200, 300]<br>'max_depth': [None, 5, 10, 15]<br>'min_samples_split': [2, 5, 10]<br>'min_samples_leaf': [1, 2, 4]<br>'max_features': ['sqrt', 'log2']
XGBOOST	'n_estimators': [100, 200, 300]<br>'max_depth': [3, 5, 7]<br>'learning_rate': [0.01, 0.1, 0.2]<br>'subsample': [0.5, 0.7, 1]
LOGISTIC REGRESSION	'C': [0.01,0.1, 1, 10]<br>'penalty': ['l2']<br>'solver': ['newton-cg', 'lbfgs', 'liblinear', 'sag']<br>'class_weight': [None, 'balanced']

Table S3 Pan-cancer classification metrics for the most sensitive model. 95% Confidence intervals are shown in the brackets.

**CANCER TYPE**	Sensitivity	AUC_ROC	Stage sensitivity				
			I	II	III	IV	n.a.
BRCA	0.5472 [0.4151, 0.6792]	0.912 [0.8549, 0.9559]	0.1667 [0.0, 0.5]	0.4167 [0.1667, 0.6667]	0.5 [0.0, 1.0]	0.6452 [0.4516, 0.8065]	1 [1.0, 1.0]
CHOL	0.587 [0.4565, 0.7174]	0.9433 [0.9068, 0.9738]	0.5 [0.0, 1.0]	0.25 [0.0, 0.5]	0.8333 [0.5, 1.0]	0.6667 [0.4583, 0.8333]	1 [1.0, 1.0]
CRC	0.6522 [0.5, 0.7826]	0.9599 [0.9316, 0.9817]	0 [0.0, 0.0]	0.5 [0.0, 1.0]	1 [1.0, 1.0]	0.625 [0.4688, 0.7812]	0.75 [0.5, 1.0]
ENDO	0.5 [0.25, 0.75]	0.8733 [0.7243, 0.9823]	0.4286 [0.1429, 0.7179]	0 [0.0, 0.0]	0.75 [0.25, 1.0]	-	-
ESO	0.2667 [0.0667, 0.5333]	0.7845 [0.6534, 0.9014]	-	0 [0.0, 0.0]	0.4 [0.1, 0.7]	0 [0.0, 0.0]	0 [0.0, 0.0]
GLIO	0.6712 [0.5616, 0.7671]	0.9424 [0.9113, 0.9686]	-	-	-	-	0.6712 [0.5616, 0.7808]
HNSSC	0.6066 [0.4754, 0.7213]	0.9377 [0.9017, 0.9683]	1 [1.0, 1.0]	0.3333 [0.0, 1.0]	0.55 [0.35, 0.75]	0.6389 [0.4722, 0.8056]	-
HCC	1 [1.0, 1.0]	0.9991 [0.9966, 1.0]	NA NA	1 [1.0, 1.0]	1 [1.0, 1.0]	1 [1.0, 1.0]	1 [1.0, 1.0]
LYM	0.7 [0.5, 0.9]	0.9223 [0.8369, 0.987]	0.5 [0.0, 1.0]	1 [1.0, 1.0]	0.6 [0.2, 1.0]	0.6667 [0.3333, 1.0]	0.5 [0.0, 1.0]
MELA	0.75 [0.5714, 0.8929]	0.9709 [0.9423, 0.9909]	-	-	0 [0.0, 0.0]	0.8077 [0.6538, 0.9615]	0 [0.0, 0.0]
MM	1 [1.0, 1.0]	0.9994 [0.9963, 1.0]	-	-	-	-	1 [1.0, 1.0]
NSCLC	0.7537 [0.7067, 0.8006]	0.9641 [0.9496, 0.9775]	0.6 [0.3, 0.9]	0.6667 [0.3333, 1.0]	0.6765 [0.5, 0.8235]	0.7668 [0.7208, 0.8163]	0.875 [0.625, 1.0]
OVCAR	0.7647 [0.6863, 0.8434]	0.9661 [0.9468, 0.9819]	0.7391 [0.5652, 0.913]	0.7 [0.4, 1.0]	0.7222 [0.5833, 0.8611]	0.8438 [0.7188, 0.9688]	1 [1.0, 1.0]
PDAC	0.4651 [0.3605, 0.5698]	0.8773 [0.8226, 0.9196]	1 [1.0, 1.0]	0.5106 [0.3617, 0.6383]	0.35 [0.15, 0.55]	0.4444 [0.2222, 0.6667]	-
PRCA	0.7273 [0.4545, 1.0]	0.9826 [0.9539, 1.0]	-	-	-	0.8 [0.4, 1.0]	0.6667 [0.3333, 1.0]
RCC	0.5556 [0.2222, 0.8889]	0.9467 [0.8614, 0.9985]	-	-	-	0.5556 [0.2222, 0.8889]	-
URO	1 [1.0, 1.0]	0.9992 [0.9954, 1.0]	-	-	-	1 [1.0, 1.0]	-
Cancer	0.6799 [0.6488, 0.7089]	0.945 [0.9291, 0.9596]	0.5926 [0.463, 0.7037]	0.51 [0.42, 0.61]	0.6154 [0.5385, 0.6923]	0.7306 [0.6899, 0.7674]	0.4094 [0.3694, 0.4565]

Supplemental Material | Figures

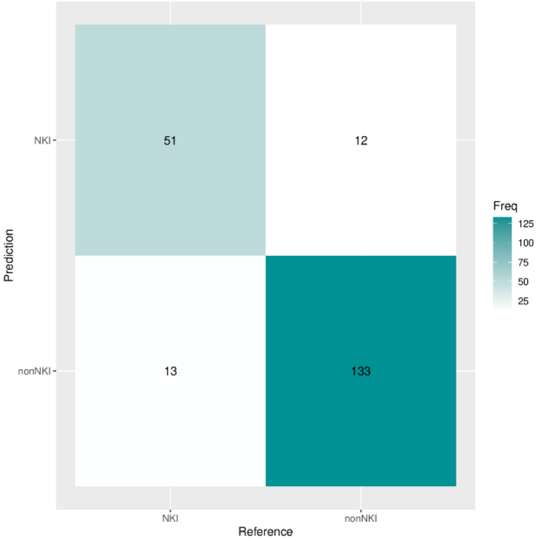

Figure S1 Confusion matrix for classification of NKI vs nonNKI NSCLC patients.

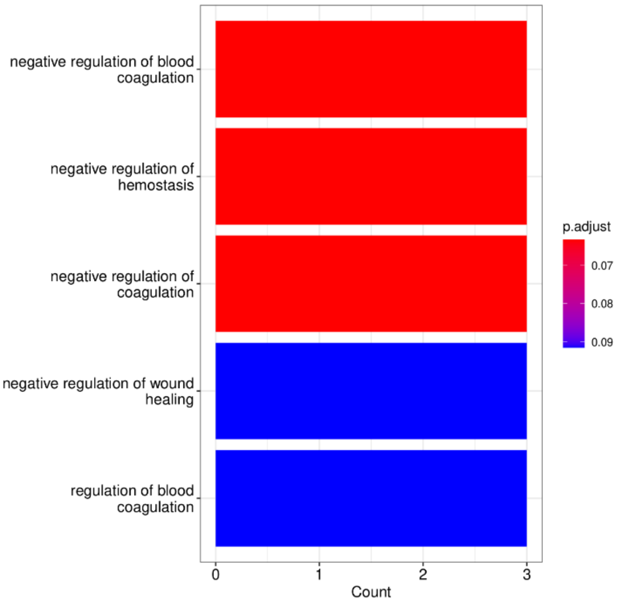

Figure S2 Gene ontology analysis of the most important features for model distinguishing NKI vs nonNKI NSCLC patients.

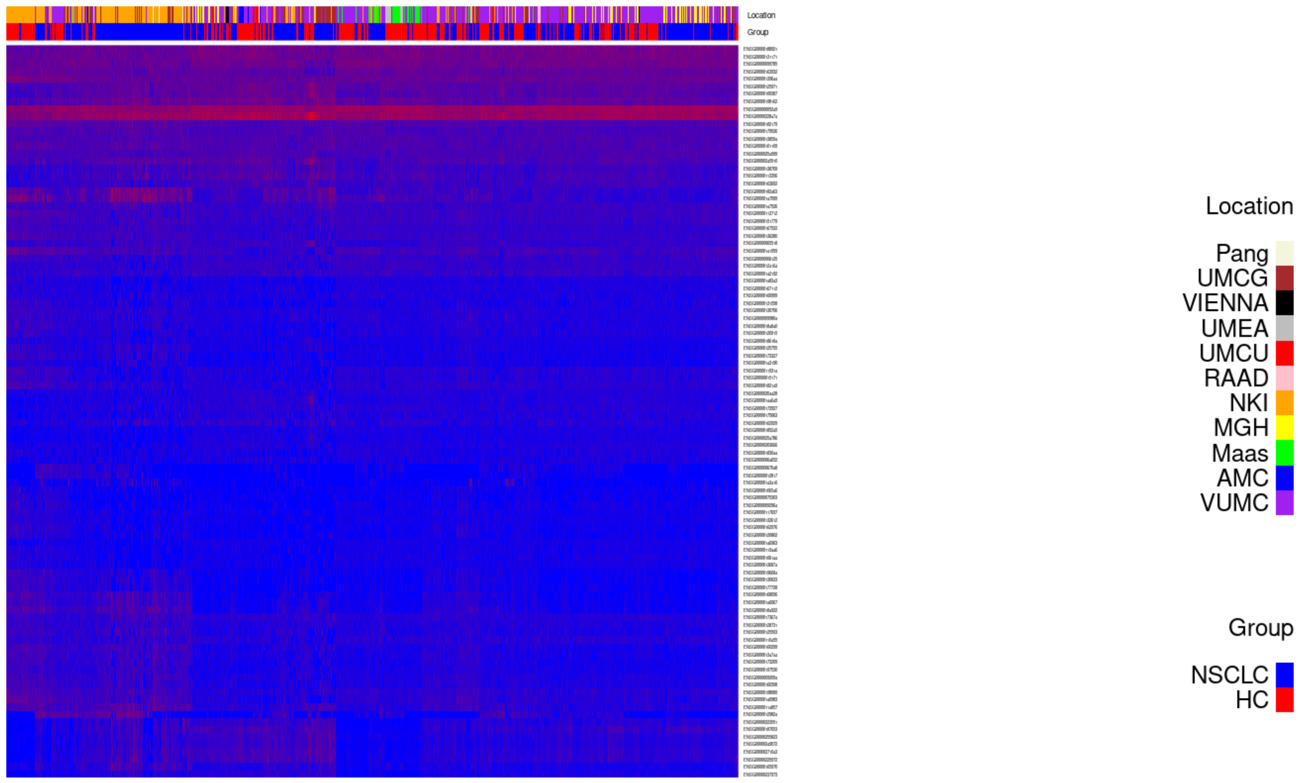

Figure S3 Heatmap showing healthy controls and NSCLC patients. The first color bar at the top depicts the institute where samples were collected. NKI is marked with orange. The second color bar shows patient groups. Transcripts used in either of the models classifying samples as NKI or non-NKI were included in the analysis.

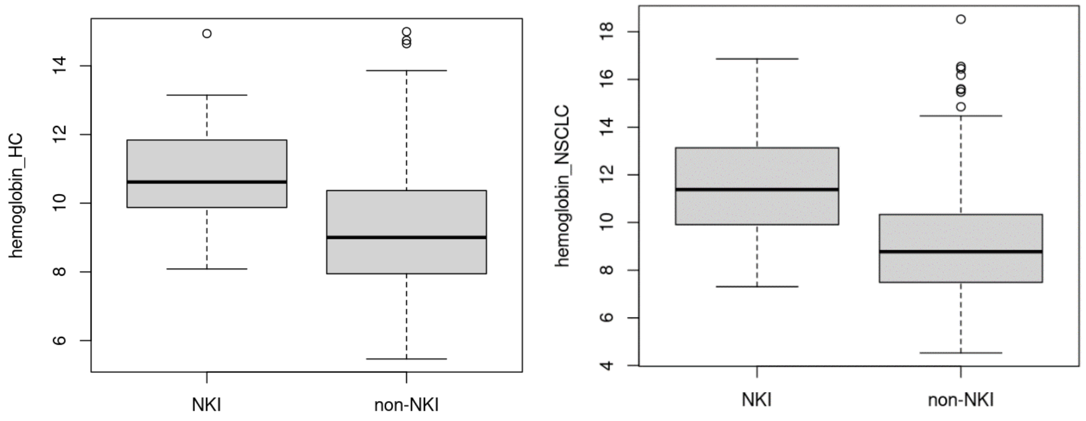

Figure S4 Comparison of hemoglobin expression levels in asymptomatically Healthy Controls and Non-Small Cell Lung Cancer based on site of sample collection.

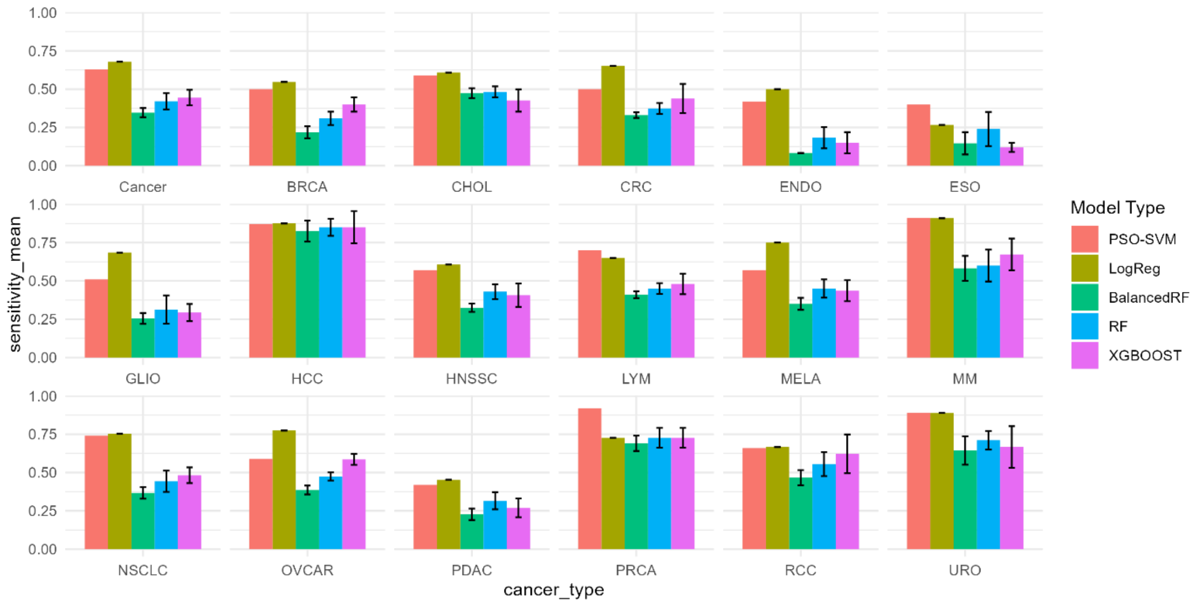

Figure S5 Sensitivity of models based on the cancer type The original PSO-SVM models' performance was added for comparison. Error bars refer to the standard deviation acquired on various random seeds. The "Cancer" class shows the overall performance in detecting cancer (binary classification Healthy vs Cancer). The rest of the diagrams show the performance across each cancer type separately.

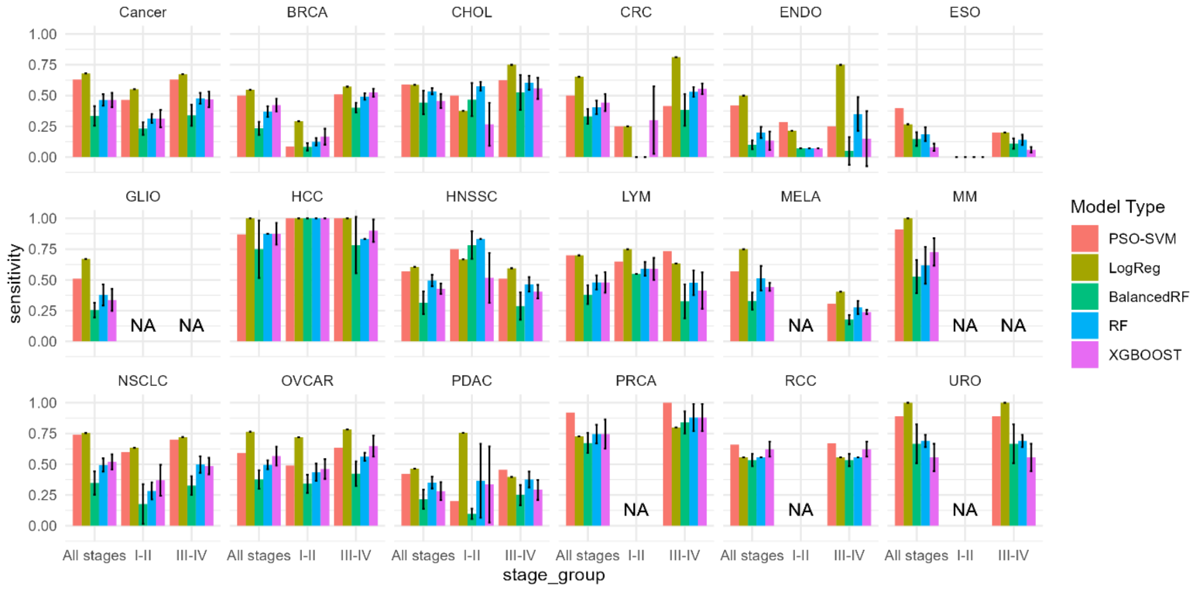

Figure S6 Sensitivity of models based on cancer type and stages. Early stages (stage I-II) and late stages (III-IV) are grouped together. Error bars refer to the standard deviation acquired on various random seeds. Cancer types that lack data from specific stages are tagged with the "NA" label. The original PSO-SVM models' performance was added for comparison. The "Cancer" class shows the overall performance across all cancer types (binary classification Healthy vs Cancer).

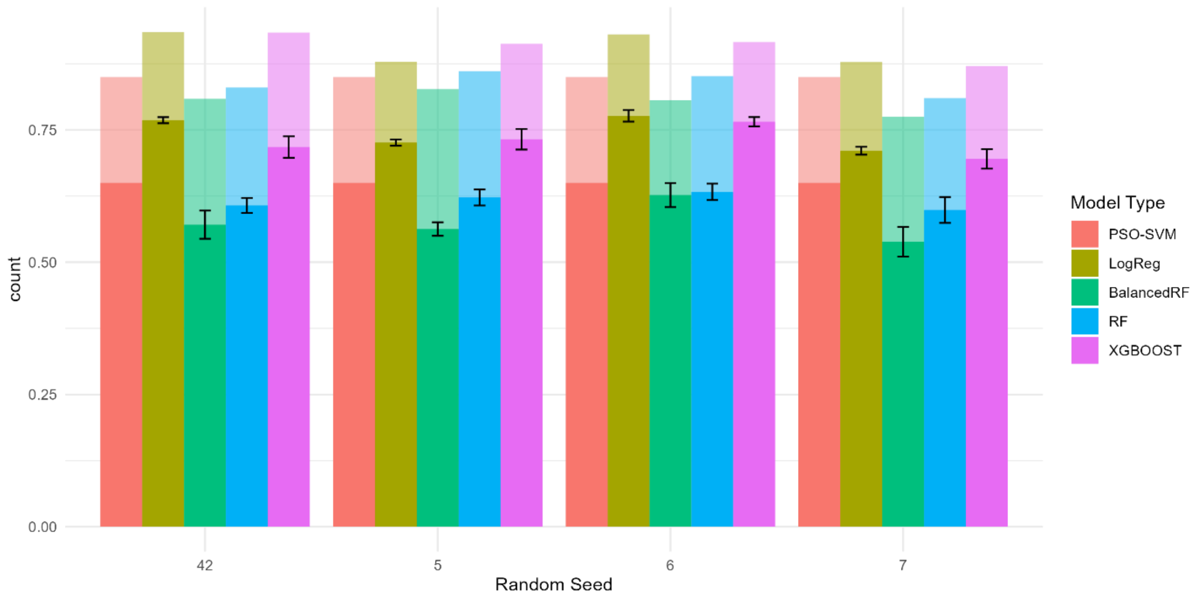

Figure S7 Detection accuracy for first prediction (dark color) and second prediction (light color) on test samples across various random seeds for multi-class models based on 5 types of cancer. Error bars refer to the standard deviation acquired from all folds. The performance of the original PSO-SVM model is added for comparison.

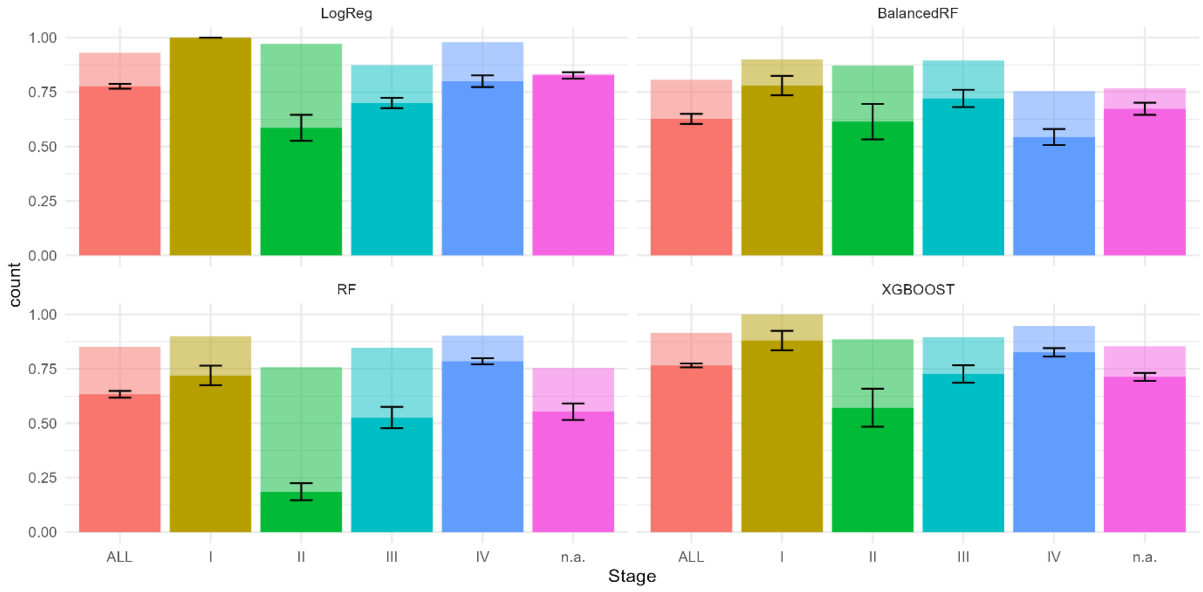

Figure S8 Detection accuracy for first prediction (dark color) and second prediction (light color) on test samples across all cancer stages for multi-class models based on 5 types of cancer. Error bars refer to the standard deviation acquired from all folds.

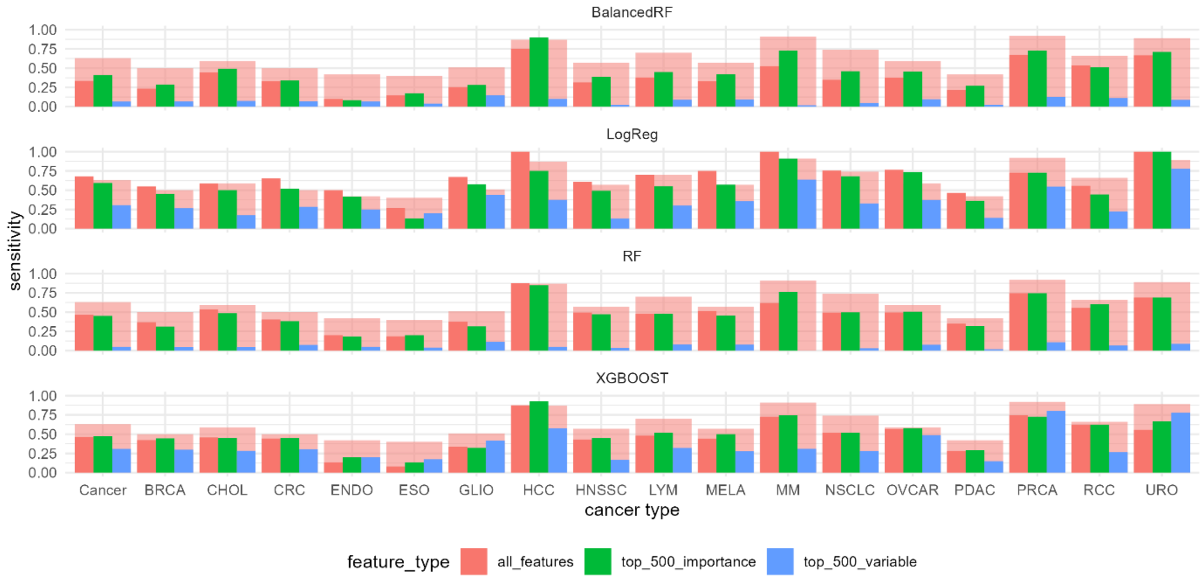

Figure S9 Sensitivity of models based on cancer type and feature group. The performance of the original PSO-SVM model trained on all available features is shown behind bars (light red) for comparison. The “Cancer” class shows the overall performance across all cancer types (binary classification Healthy vs Cancer).

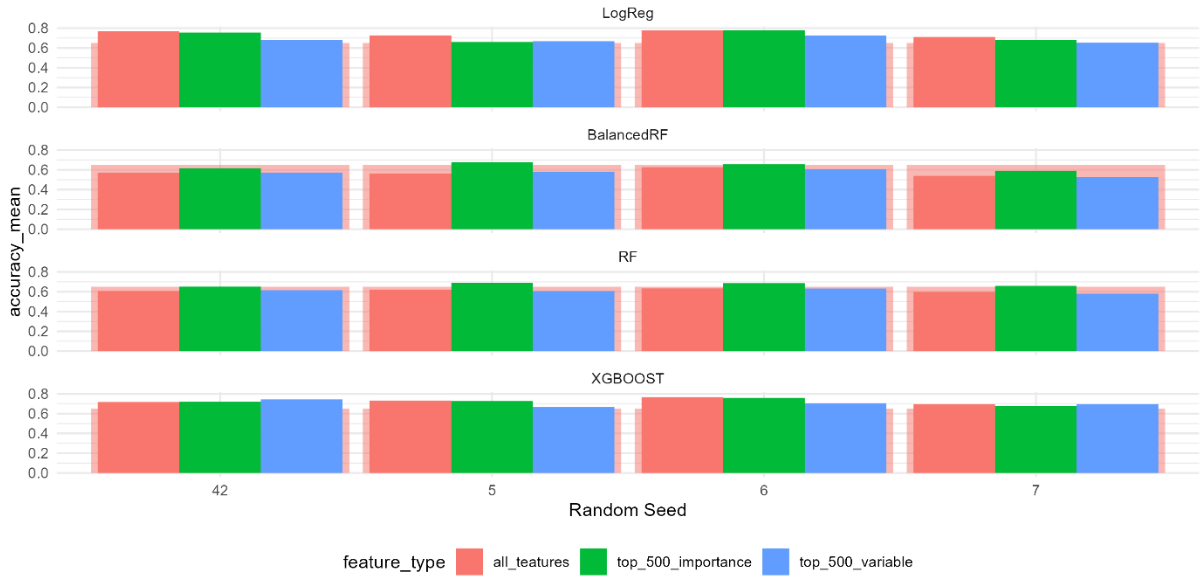

Figure S10 Detection accuracy for the first prediction across various random states for multi-class models based on 5 types of cancer. The performance of the original PSO-SVM model trained on all available features is shown behind bars for comparison.
